# The influence of cultivation conditions on the formation of psychoactive salvinorin A, salvinorin B, rosmarinic acid and caffeic acid in *Coleus scutellarioides*

**DOI:** 10.1038/s41598-024-57399-y

**Published:** 2024-03-20

**Authors:** Maciej Jakobina, Jacek Łyczko, Antoni Szumny, Renata Galek

**Affiliations:** 1 Department of Genetics, Plant Breeding and Seed Production, Faculty of Life Sciences and Technology, Wrocław University of Environmental and Life Sciences, Grunwaldzki Square 24A, 50-363 Wrocław, Poland; 2 Department of Food Chemistry and Biocatalysis, Faculty of Biotechnology and Food Science, Wrocław University of Environmental and Life Sciences, Norwida 25, 53-375 Wrocław, Poland

**Keywords:** Chemical modification, Biochemistry, Plant sciences

## Abstract

*Coleus scutellarioides* (L.) Benth. is a popular species in the world, known for its characteristic magnificent colourful leaves. The study has revealed that the contents of rosmarinic acid and caffeic acid are significantly higher in the plant tissues cultivated in vivo than when under in vitro conditions. The performed qualitative and quantitative analyses confirmed the presence (whose averaged content) of salvinorin A (6.65 µg/1 g of fresh plant) and salvinorin B (50.46 µg/1 g of fresh plant) in tissues of *Coleus scutellarioides* (L.) Benth. of ‘Electric lime’ variety. The greatest quantities of these compounds were recorded for plants cultivated in vitro on the MS medium enriched with NAA (naphthyl-1-acetic acid) at a concentration of 0.5 mg∙ dm^–3^. The research detected differences in the amounts of compounds between plants grown in vivo and those cultivated in vitro. Addition of plant growth regulators into the breeding medium under in vitro conditions was found affecting the amounts of compounds in plant tissues.

## Introduction

**Figure 1:**
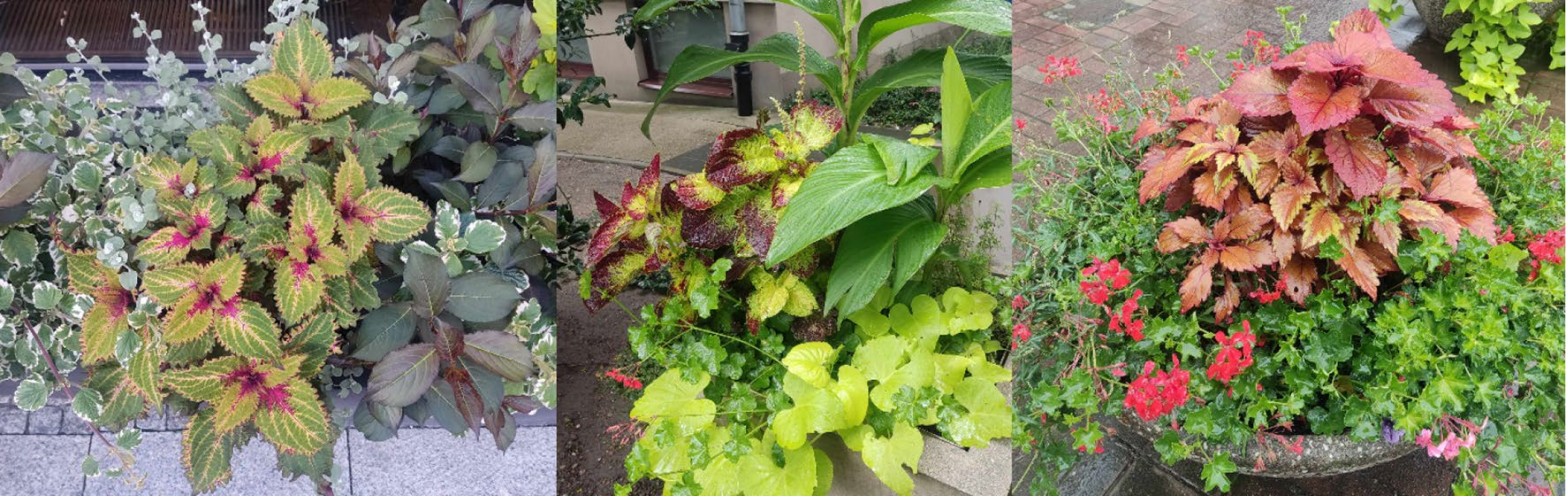
*Coleus scutellarioides* in municipal flower-beds*.* (Photo: M. Jakobina).

The coleus *Coleus scutellarioides* (L.) Benth., commonly referred to as the painted nettle, represents the genus *Coleus* ranked within the family *Lamiaceae* Lindl.^1^. The native distribution area of *Coleus scutellarioides* (L.) Benth. is the territory of tropical and subtropical Asia, reaching northern Australia. It is frost-resistant up to the USDA 10–11 zone. In other parts of the globe it is cultivated outdoors as an annual or indoors as a perennial plant. It is a widely-spread and well-known plant among gardeners owing to its decorative and ornamental leaves. This species is often found within flower-beds in places open to the public (Fig. 1)^2,3^.

*Coleus scutellarioides* possesses medicinal potential^4–9^. It was the first species to be used for acquisition of rosmarinic acid in large quantities^10,11^.

The psychoactive properties of *Coleus scutellarioides* are controversial. Sources record occurrence of psychoactive effects during leaf burning. The effect can be achieved via burning of as few as three leaves. *Coleus scutellarioides* was used by Maztec Indians, inhabiting southern Mexico, as a substitute for the seer's sage *Salvia divinorum*, another representative of the *Lamiaceae*, which contains psychoactive salvinorin A. However, not all investigations confirm that *Coleus scutellarioides* acts as a psychoactive agent^12–15^.

Although reports on euphoria experienced under the influence of *Coleus scutellarioides* are scant, one can still find information that the species possesses similar properties as *Salvia divinorum*. There is a record about an over twenty-year-old male who came to the emergency ward with a psychotic episode after consuming *Coleus scutellarioides*. He had learned from social media that by chewing leaves of this plant he could elicit in himself a state of euphoria. The comprehensive examination of this patient excluded the influence of other factors upon his state. Therefore, this case draws attention to *Coleus scutellarioides* as a potential psychotropic drug, which requires clinical consideration and further research^16^.

So far there has been no records in the literature about identified psychoactive substances occurring in *Coleus scutellarioides.* And that is why an attempt was undertaken to perform detailed analyses with a view to detect presence of psychoactive compounds in a strictly specified variety of *Coleus scutellarioides.*

## Results

**Figure 2:**
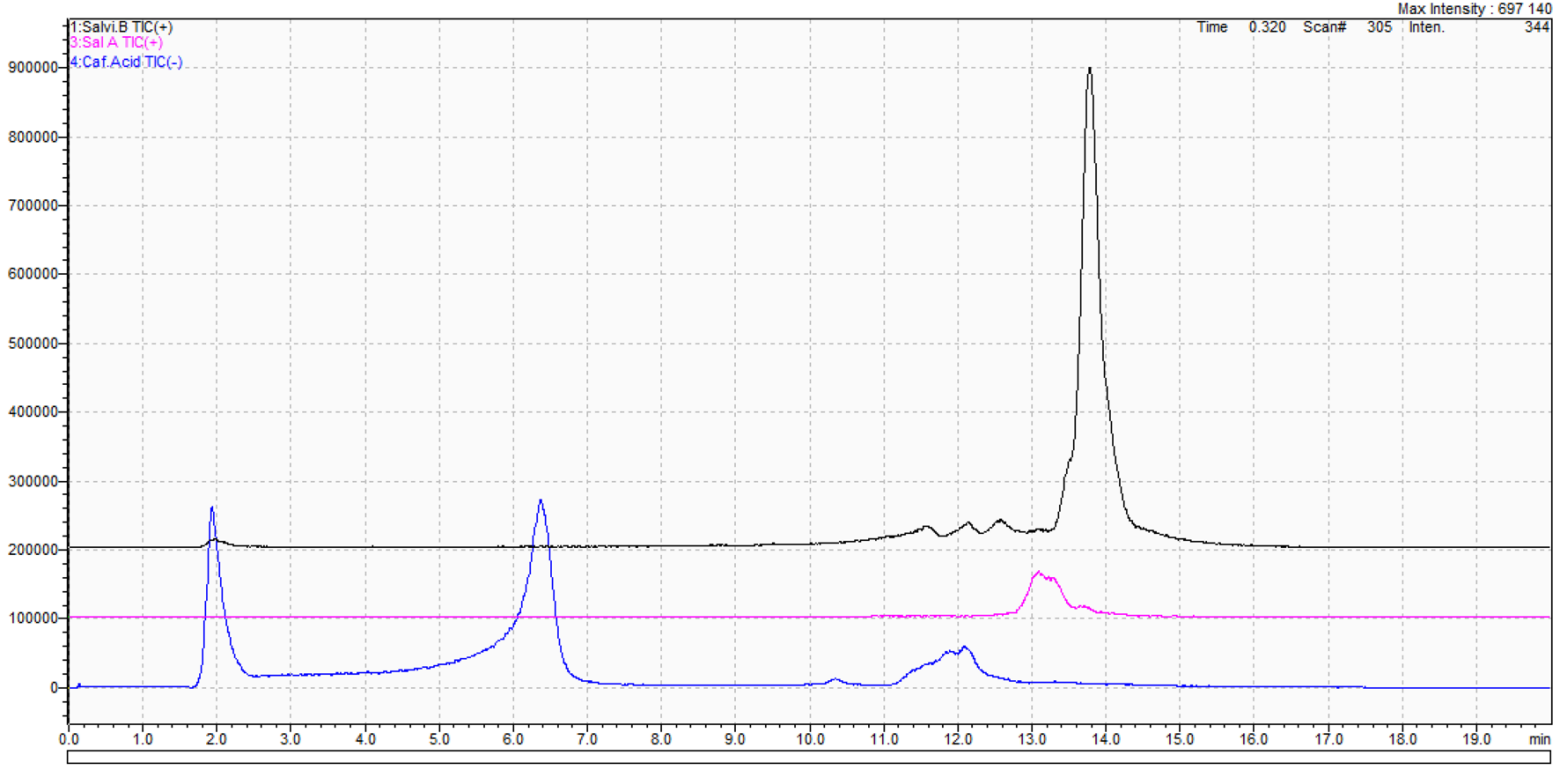
Chromatogram of salvinorin A (pink), salvinorin B (black) and caffeic acid (blue).

**Figure 3:**
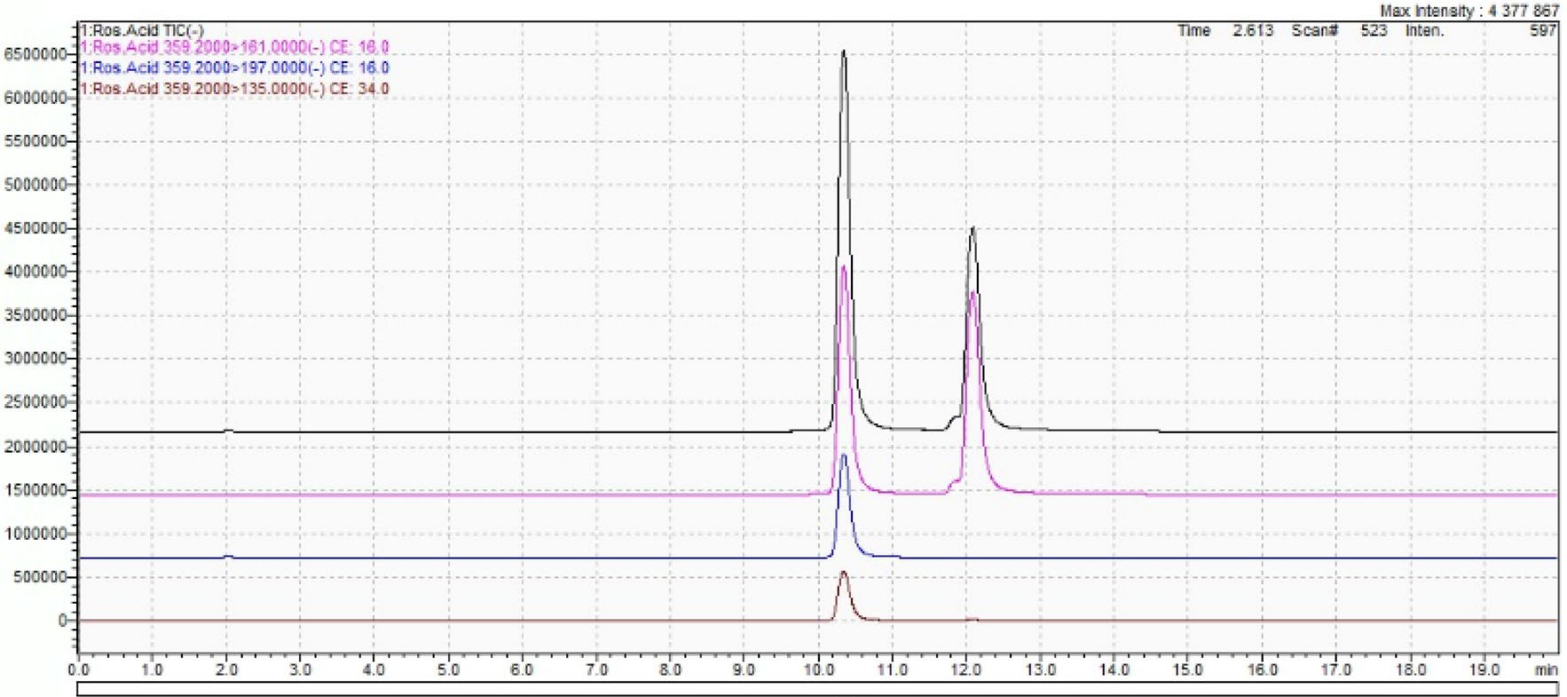
Chromatogram of rosmarinic acid.

The qualitative analysis of the contents of all the four compounds, i.e. salvinorin A, salvinorin B, caffeic acid and rosmarinic acid, successfully confirmed their occurrence in the tissues of *Coleus scutellarioides* (L.) Benth. variability 'Electric lime'. Quantitative analysis was carried out for salvinorin A, salvinorin B, caffeic acid (Fig. 2) and rosmarinic acid (Fig. 3).

**Diagram 1:**
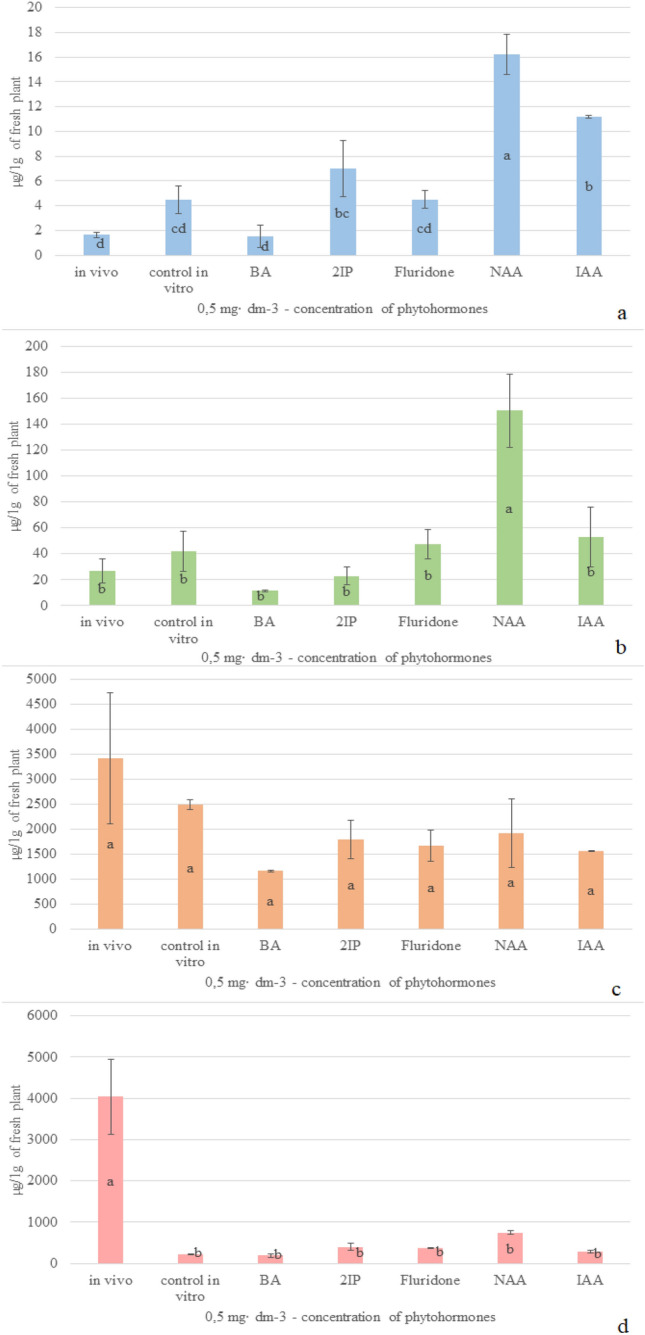
Impact of cultivation conditions on the contents of analyzed compounds in fresh tissue: (**a**) salvinorin A; (**b**) salvinorin B; (**c**) caffeic acid; (**d)** rosmarinic acid.

Plant growing conditions (in vivo or in vitro) as well as supplementing the breeding medium with growth regulators when under in vitro conditions significantly affect the contents of particular compounds in plant tissues (Diagram 1a–d). The highest content of salvinorin A (Diagram 1a), calculated in relation to the salvinorin B content, was found in plant tissues cultivated in vitro on the medium enriched with NAA – over three times exceeding the values obtained for plants bred on the medium without phytohormones. On the BA medium and under in vivo conditions the content of salvinorin A was over three times lower than in plants grown on the control medium. The greatest content of salvinorin B (Diagram 1b) was determined in the tissues of plants enriched with NAA – over three times higher than in plants cultivated without addition of phytohormones. The plants bred on the medium supplemented with BA were characterized by over three times lower content of salvinorin B as compared with the control medium. Similar levels of salvinorin B were found in plants cultivated in vivo and under in vitro conditions on the medium containing no phytohormones. The highest content of caffeic acid (Diagram 1c) was established to occur in plants coming from in vivo cultivation. The content of this compound exceeded 1,4 times the values obtained for the control medium in vitro. In plants bred in vitro on the medium enriched with BA the content of caffeic acid was over twice lower than in the case of plants growing on the medium without phytohormones. The highest amount of rosmarinic acid was revealed in plants cultivated in vivo – twenty times more as much in comparison with plants under in vitro conditions (Diagram 1d). Comparing with the control medium without phytohormones, the lowest content of this compound was detected in plants supplemented with BA.

Summing up, the highest mean values of the analyzed compounds were recorded for plants cultivated under in vivo conditions, i.e. 1871.39 µg ∙ 1 g^–1^ of fresh tissue, while the lowest contents in plants bred in vitro and supplemented with BA – 341.98 µg ∙ 1 g^–1^ of fresh tissue.

## Discussion

Rosmarinic acid exhibits an array of interesting biological effects. More comprehensive knowledge of this acid could contribute to its proper use in treatment and prevention of a wide range of health ailments^17–21^.

In plants rosmarinic acid should act as a pre-formed constitutively assembled defense compound. Rosmarinic acid is easily accumulated in undiffentiated plant cell cultures, in certain cases at concentrations highly exceeding those recorded for the plant itself. The first plant which was employed for production of rosmarinic acid in cell culture was *Coleus scutellarioides*^10,11^.

There is one publication which compares three varieties of *Coleus scutellarioides* with respect to the content of rosmarinic acid in plants grown in vivo. This research compares the following varieties: 'Defiance', 'Shocking Pink' and 'Small Leaf'. Based on thin-layer chromatography, a conclusion has been reached that variety 'Small Leaf' may have the highest content of rosmarinic acid as related to the others^22^.

Plants representing the family *Lamiaceae* are characterized by a high content of rosmarinic acid. For instance, *Mellisa officinalis* contains 36.5 mg∙ 1 g^–1^ of rosmarinic acid, *Mentha piperita* – 28.2 mg∙ 1 g^–1^, *Mentha spicata* – 58.5 mg∙ 1 g^–1^ whereas *Rosmarinus officinalis* only 7.2 mg∙ 1 g^–1^^23^. Increasing the biosynthesis of rosmarinic acid in *Coleus scutellarioides* has been an object of investigations undertaken by numerous research teams. Application of the combination IBA (indolylbutyric acid), BAP (6-benzylaminopurine) and GA_3_ (gibberellic acid) (0.5 mg∙ dm^–3^) induced the highest efficiency of the rosmarinic acid production, amounting to 65.2% in in vitro cultures of whole plants^24^. Improvement in the biosynthesis of rosmarinic acid under in vitro plant cultures has been attained in the common sage *Salvia officinalis* L. via supplementation of the breeding medium with phytohormones: in these experiments IAA (indole-3-acetic acid) combined with BAP (6-benzylaminopurine) was added^25^.

Caffeic acid possesses a potential applicable in cosmetology and medicine thanks to its anti-oxidant, anti-microbial and also anti-cancer activity^26–29^. The content of caffeic acid in the roots of *Asparagus officinalis* amounts to 1.7–3.0 mg∙ 1 g^–1^ whereas in *Ocimum basilicum* it reaches up to a level of 0.83 mg · 1 g^–1^^12,30^. Effective growth of the caffeic acid content has been achieved in tissue cultures of the Eastern purple coneflower *Echinacea purpurea* L. Moench. through application of the combination GA_3_ (gibberellic acid), paclobutrazole and uniconazole^31^.

The content of salvinorin A in dry leaves of *Salvia divinorum* of different origin can reach from 55.75 to 163.97 μg · 100mg^–1^ dry leaves. Employment of the DART-HRMS method shows that normally the content of salvinorin A amounts to 1.54 mg · 1 g^–1^ raw leaves, whereas in improved extracts salvirnorin A is present at a level of 13.0–53.2 mg · 1 g^–1^ raw leaves. In Mexico research on legal drugs determined the salvinorin A range from 8.32 to 56.52 mg · 1 g^–1^ dried leaf in the species mentioned. Investigators have established that 200–500 μg of salvinorin A induces strong hallucinogenic effects lasting for 20–30 min^32–35^. Salvinorins A and B represent diterpenes of neo clerodane. Salvinorin A is a hallucinogen occurring naturally in *Salvia divinorum*. With regard to strength it competes with a synthetic hallucinogen, namely diethylamide of lysergic acid. Salvinorin A is a typical non-nitrogenous selective antagonist of the opioid kappa receptor. Depending on the selectivity of salvinorin A for the opioid kappa receptor, this receptor constitutes a potential molecular target for the development of pharmaceuticals used for the study of characteristic features of different perception disorders, including schisophrenia, the Alzheimer's disease and bipolar affective disorder^36^. It possesses therapeutic potential in the treatment of pain, mood and personality disorders, substance abuse and gastro-intestinal disorders, which implies that non-alkaloids can be a potential scaffolding for the development of medicaments for the receptors coupled with aminergic proteins G^37^.

Salvinorin B is a strong and selective analogue of the salvinorin A opioid kappa receptor. It is characterized by pro-depressant, anti-cocaine activity. However, in antinociceptive tests, this compound was not found acting as strongly as salvinorin A in reducing pain^38^.

Derivatives of salvinorin A and salvinorin B are salvianic acid A and salvianic acid B, a biosynthetic increse of which has been attained through supplementation of the breeding media with ABA (abscisic acid) in tissue cultivation of the red sage *Salvia miltiorrhiza* Bunge^39^.

Metabolic engineering is aimed at creation of new compounds in an organism, improvement in the production of the present compounds or mediation in the degradation of compounds^40^. Investigations carried out yield a conclusion that the metabolic pathways should be studied in the context of a whole plant and not only of a single cell. Even the simplest modifications can lead to induction of changes in the entire organism. At present the commonest strategy of targeted metabolic engineering is modification of a gene or genes, study of their expression and control of enzymes involved in the metabolic pathways^41–43^. Yet, this is not the only means to modify the metabolic pathways in plants. So far numerous biosynthetic pathways in plant compounds have been successfully modified with the application of plant growth regulators^44,45^. Determination and understanding of the mechanism of plants' reaction to phytohormones and identification of specific genes undergoing expression under supplementation will be of key importance in the future planning of improvement or modification of metabolic pathways in the biosynthesis of plant compounds.

The precursor of salvinorins is geranylgeranyl diphosphate (GGPP), the biosynthesis of which occurs via the 2-C-methyl-erythritol 4-phosphate (MEP) pathway^46,47^. 6-benzyloaminopurine reduce the amount of compounds witch are biosynthesized on the MEP pathway^48^, similar as in our case. The other studies indicate the influence of NAA on the MEP pathway^49,50^. Our results indicate that this plant growth regulator stimulated the biosynthesis of identificed secondary metabolites. Fluridone is commonly known to interfere with carotenoid and chlorophyll biosynthesis by modifying the MEP pathway^51,52^. Caffeic acid is one of the key intermediates of the plant phenylpropanoid pathway^53,54^. Rosmarinic acid biosynthesis is related to the phenylpropanoid pathway and the shikimic acid pathway^55^. It was proven that it was early steps of the phenylpropanoid pathway are regulated by auxins^56^. Moreover, the influence of cytokines on the discussed pathway was demonstrated^57^. Auxins are synthesized from L-tryptophan which is produced on the shikimate pathway^58^. Thus, the addition of auxins modifies this pathway, however, there are insufficient studies describing this effect. The effect of cytokinins has not been sufficiently studied so far. Generaly plant hormones have a comprehensive impact on the biosynthetic pathways of secondary metabolites in plants. Which is also confirmed by our results when using chemical compounds: BA, 2IP, fluridone, NAA and IAA.

## Conclusions

The hitherto conducted investigations concentrated mainly on analysis of compounds contained in *Coleus scutellarioides* (L.) Benth. without specification of the variety studied. This research has revealed that specifically in *Coleus scutellarioides* variety 'Electric lime' the contents of rosmarinic and caffeic acids are significantly higher in tissues of plants cultivated under in vivo conditions. What is more, the performed qualitative and quantitative analyses confirm the presence of salvinorin A and salvinorin B in *Coleus scutellarioides* (L.) Benth. The presently discussed is the first successful attempt at such confirmation. And thus, this finding corroborates the reports indicating presence of psychoactive compounds in the species under analysis.

The present experiments detected differences in the amounts of the compounds concerned relative to the mode of cultivation, which is whether under in vivo or in vitro conditions. Supplementation of the breeding media with phytohormones under in vitro conditions was found affecting the percentage ratio and contents of particular compounds in plant tissues. Thereby, plant growth regulators have been proven to possess a potential for inducing modifications in the biosynthesis of compounds in plants.

The common occurrence of *Coleus scutellaroides* (L.) Benth., whose tissues contain salvinorins, poses a threat to humans since these psychoactive compounds can be dangerous to health, and that is why they should not be easily available.

## Methods

### Research material and conditions of in vitro culture

**Figure 4:**
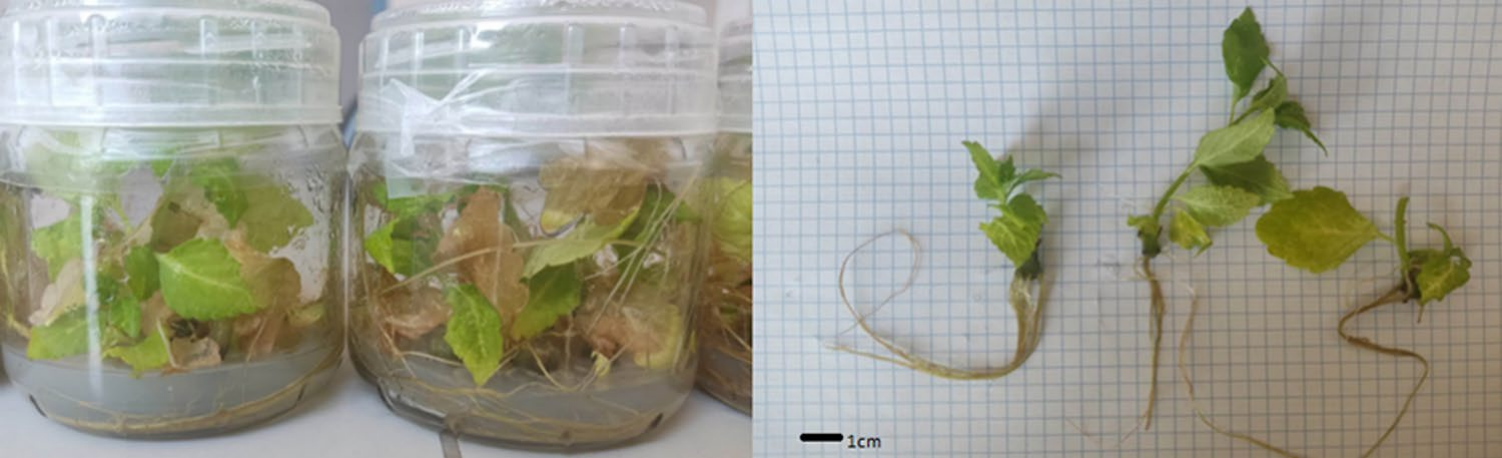
In vitro cultivation of the parent plant of *C. scutellarioides (L.)* Benth. representing 'Electric lime' variety (Photo: M. Jakobina).

The initial material was composed of above-ground parts of *Coleus scutellarioides* (L.) Benth. plants representing the 'Electric lime' variety, coming from supermarket. For inducing in vitro cultures, fragments of stems with nodes were used. The prepared material was put onto the MS medium (Murashige & Skoog^59^) at pH = 5.8. The plants were micropropagated on the MS medium without growth regulators (Fig. 4).

Experimental material were microplantlets, being leafless nodal explants, and apical meristems. The cultivation was carried out on the MS medium with five plant growth regulators, namely 0.5 mg∙ dm^–3^ BA (6-benzylaminopurine), 0.5 mg∙ dm^–3^ 2iP (2-isopentyladenine), 0.5 mg∙ dm^–3^ IAA (indole-3-acetic acid), 0.5 mg∙ dm^–3^ NAA (1-naphthalene acid), 0.5 mg∙ dm^–3^ fluridone as well as on an MS control medium without addition of phytohormones. Afterwards the plants were subjected to chemical analysis.

### Quantification of compounds by the LC–MS/MS technique

The plant material constituted leaves and stems coming from plants bred on varied substrata under in vitro conditions and also from plants cultivated in vivo. The initial plant used for inducing in vitro cultures was homogenized by grinding in a mortar in liquid nitrogen, after which 1 g of the homogenate was macerated in 2 ml of 96% ethanol. The material was vortexed and subsequently transported into the ultrasonic bath for 20 min. The samples were kept in darkness for 18 h due to photolability of compounds and improved extraction efficiency. Then, the material was vortexed again and placed into the ultrasonic bath for 20 min. The samples were centrifuged for 10 min at 12,000 RPM and collection of the extract was carried out. To the remaining plant material 2 ml of pure methanol was added, after which the material was vortexed, transferred to the ultrasonic bath for 20 min, centrifuged for 10 min at 12,000 RPM and subsequently the extract was collected. The procedure was repeated until the moment when 6 ml of extract was obtained. The extract was divided into two samples. Through a filter (PTFE 0.22 µm), the extract was transferred to round bottom flasks, and evaporated using a vacuum evaporator at room temperature (SAVANT SPD121P SpeedVac Concentrator The dry remnant was dissolved in 1 ml of acetonitrile, then transferred for 20 min to the ultrasonic bath in order to make sure that the extract got entirely dissolved in ACN. This was followed by centrifugation for 5 min at 13,000 RPM. Then, 250 µl of the solution was poured into a vial of a volume of 2 ml, to which 500 µl of acetonitrile was added. The quantitative analysis of salvinorin B, salvinorin A (as related to the amount of salvinorin B), caffeic acid and rosmarinic acid was performed on an LCMS 8045 (Shimadzu, Kyoto, Japan) equipped with the C18 Kinetex (2.6u, 100A, 100 × 3.0 mm, Phenomenex, Torrance, CA, USA) column. The analysis was conducted in two replications. Chromatographic separation for quantitative determination of the contents of salvinorin B, rosmarinic acid and caffeic acid was performed with the use of two mobile phases: water with addition of 0.1% [v/v] formic acid (A and acetonitrile (B). The following gradient programme was used for analytes separation: initially 10% of B, then 20% B in 5th min, then 60% B in 10th min, then 10% B in 13th min and kept up to 20th min. The flow rate was set at 0.3 ml/min, column oven was set for 35 °C while the injection was set at 25.0 µl for salvinorin B and caffeic acid, and at 5.0 µl for rosmarinic acid quantification. Mass spectrometry was carried out with ionization through electrospraying and mode MRM (Table 1):

**Table 1:** Parameters of mass spectrometry.

Positive ionization		
Salvinorin A		
433.0 > 373.0 m/z		
Q1 Pre Bias [V] – 20.0		
CE – 17.0		
Q3 Pre Bias [V] – 24.0		
Salvinorin B		
391.0 > 373.05 m/z	391.0 > 251.1 m/z	391.0 > 145.05 m/z
Q1 Pre Bias [V] – 20.0	Q1 Pre Bias [V] – 20.0	Q1 Pre Bias [V] – 20.0
CE -10.0	CE -18.0	CE -36.0
Q3 Pre Bias [V] – 24.0	Q3 Pre Bias [V] – 24.0	Q3 Pre Bias [V] – 24.0
Negative ionization		
Rosmarinic acid		
359.2 > 161.0 m/z	359.2 > 197.0 m/z	359.2 > 135.0 m/z
Q1 Pre Bias [V] 17.0	Q1 Pre Bias [V] 17.0	Q1 Pre Bias [V] 17.0
CE 16.0	CE 16.0	CE 34.0
Q3 Pre Bias [V] 18.0	Q3 Pre Bias [V] 19.0	Q3 Pre Bias [V] 26.0
Caffeic acid		
179.4 > 135.05 m/z	179.4 > 134.05 m/z	179.4 > 107.0 m/z
Q1 Pre Bias [V] 11.0	Q1 Pre Bias [V] 11.1	Q1 Pre Bias [V] 11.1
CE 17.0	CE 30.0	CE 22.0
Q3 Pre Bias [V] 27.0	Q3 Pre Bias [V] 24.0	Q3 Pre Bias [V] 20.0

At given parameters: the flow of the sprayed gas was 3 L/min, flow of the heating and drying gas – 10 L/min, interphase temperature – 300 °C, desolvation temperature – 526 °C, temperature of the semiconductor laser – 250 °C, of the heating block – 400 °C.

Quantitative determination of salvinorin B, rosmarinic acid and caffeic acid in *Coleus scutellarioides* (L.) Benth. variability 'Electric lime' was performed in comparison with the pure model and the calibration curve whereas the quantification of salvinorin A was calculated as related to the content of salvinorin B. Qantitative analysis was performed based on the external standard method.

### Statistical analysis

The results were expressed as mean measurements and recorded as a mean value. One-factor variance analysis (ANOVA) was carried out in order to verify lack of significant effect of plant growth regulators applied in in vitro cultures on the chemical compostion of *Coleus scutellarioides* (L.) Benth. 'Electric lime'. The hypothesis assumed no influence of the used breeding medium on a plant's chemical compostion. Significant differences were estimated at a level of 0.05 and 0.01. In cases when variance analysis yielded a significant result, the Tukey honestly significant difference (HSD) test was employed to compare the values^59^. The outcome underwent statistical analysis with the help of the Statistica programme in version 13.3.

### Statement

All plant experiments were conducted in accordance with institutional, national and international guidelines and regulations.

## Data Availability

The datasets used and/or analysed during the current study available from the corresponding author on reasonable request. All relevant data are within the paper.
