# Author Correction: The influence of cultivation conditions on the formation of psychoactive salvinorin A, salvinorin B, rosmarinic acid and caffeic acid in *Coleus scutellarioides*

**DOI:** 10.1038/s41598-024-65402-9

**Published:** 2024-06-24

**Authors:** Maciej Jakobina, Jacek Łyczko, Antoni Szumny, Renata Galek

**Affiliations:** 1 Department of Genetics, Plant Breeding and Seed Production, Faculty of Life Sciences and Technology, Wrocław University of Environmental and Life Sciences, Grunwaldzki Square 24A, 50-363 Wrocław, Poland; 2 Department of Food Chemistry and Biocatalysis, Faculty of Biotechnology and Food Science, Wrocław University of Environmental and Life Sciences, Norwida 25, 53-375 Wrocław, Poland

Correction to: *Scientific Reports* 10.1038/s41598-024-57399-y, published online 20 March 2024

The original version of this Article contained a repeated error where the species name ‘*Coleus scutellarioides* (L.) Benth.’ was incorrectly given as ‘*Coleus scutellarioides* (L.) Benh.’ in the Abstract, in the first paragraph of the Results, in the Conclusions, in the Methods under the subheadings ‘Research material and conditions of in vitro culture’ and ‘Quantification of compounds by the LC–MS/MS technique’, respectively, and lastly, in the Statistical analysis.

The original Article has been corrected.

